# Future Possible Changes in Medically Underserved Areas in Japan: A Geographic Information System-Based Simulation Study

**DOI:** 10.3390/jmahp12020010

**Published:** 2024-06-03

**Authors:** Akihisa Nakamura, Eiji Satoh, Tatsuya Suzuki, Soichi Koike, Kazuhiko Kotani

**Affiliations:** 1 Division of Community and Family Medicine, Center for Community Medicine, Jichi Medical University, Shimotsuke City 329-0498, Japan; m06066an@jichi.ac.jp; 2 Department of Architecture and Urban Design, School of Regional Design, Utsunomiya University, Utsunomiya City 321-8585, Japan; e-satoh@cc.utsunomiya-u.ac.jp; 3 Program in Architecture, Civil and Environmental Engineering, Department of Engineering and Design, Kagawa University, Takamatsu City 761-0396, Japan; suzuki.tatsuya@kagawa-u.ac.jp; 4 Division of Health Policy and Management, Center for Community Medicine, Jichi Medical University, Shimotsuke City 329-0498, Japan

**Keywords:** accessibility, institutional closure, healthcare policy, geographic information system, rural health

## Abstract

Background: A decrease in populations could affect healthcare access and systems, particularly in medically underserved areas (MUAs) where depopulation is becoming more prevalent. This study aimed to simulate the future population and land areas of MUAs in Japan. Methods: This study covered 380,948 1 km meshes, 87,942 clinics, and 8354 hospitals throughout Japan as of 2020. The areas outside a 4 km radius of medical institutions were considered as MUAs, based on the measure of areas in the current Japanese Medical Care Act. Based on the population estimate for a 1 km mesh, the population of mesh numbers of MUAs was predicted for every 10 years from 2020 to 2050 using geographic information system analysis. If the population within a 4 km radius from a medical institution fell below 1000, the institution was operationally assumed to be closed. Results: The number of MUAs was predicted to decrease from 964,310 (0.77% of the total Japanese population) in 2020 to 763,410 (0.75%) by 2050. By 2050, 48,105 meshes (13% of the total meshes in Japan) were predicted to be new MUAs, indicating a 31% increase in MUAs from 2020 to 2050. By 2050, 1601 medical institutions were tentatively estimated to be in close proximity. Conclusions: In Japan, the population of MUAs will decrease, while the land area of MUAs will increase. Such changes may reform rural healthcare policy and systems.

## 1. Introduction

Although the global population is increasing, some countries are experiencing a decrease in their total population [1]. The populations of 55 countries and areas are projected to decrease by 1% or more between 2019 and 2050 [1]. For instance, the populations of Japan, Italy, and Spain are expected to decrease by approximately 19%, 10%, and 7%, respectively [1]. The largest relative decline in population size, with losses of approximately 20% or more, are expected in countries such as Bulgaria, Latvia, and Lithuania. The main causes of the decrease in populations include sustained low levels of fertility and increased movement of people from rural to urban areas [1,2,3]. This could lead to a shrinking market in healthcare, affecting healthcare systems [3,4]. In countries with decreasing populations, depopulation is a problem in rural areas, and rural depopulation will worsen the management of medical institutions and force some to close [5,6,7,8]. This would likely be the case in rural areas with limited healthcare services, that is, medically underserved areas (MUAs) [5,6,7,8]. The closure of a healthcare institution indicates impaired access to healthcare for its neighboring populations, leading to the creation of new MUAs [5,6,7]. Ensuring healthcare access and systems in MUAs is thus a concern in countries with decreasing populations [3,4].

The effects of depopulation on MUAs are an issue in Japan, where the population is predicted to decrease, by approximately 19%, from 125 million in 2020 to 101 million by 2050 [9,10]. Under the current conditions in Japan, a decrease in healthcare access for those living in MUAs may be inevitable [9,10]. In rural healthcare systems in Japan, “non-physician community” (NPC) (mu-ichiku in Japanese) is a policy term that implies an MUA [11,12,13]. Under this policy, an NPC has been defined as a place that has ≥50 residents within a 4 km radius with difficult access to medical institutions and physicians [11,12,13]. The NPC needs to be certified by the local government [11,12]. According to the current Japanese Medical Care Act, one of the requirements for establishing a rural public clinic is that there must be no medical institutions within a 4 km radius and that the population must be 1000 or more [13,14,15]. Owing to progress in ensuring physicians in NPCs and partially improving access to medical care by developing transport systems, the number of NPCs decreased from 2920 in 1966 to 557 in 2022 [11]. However, the decrease in access for those living in MUAs with the depopulation in Japan may lead to an increase in the number of NPCs in the future. Therefore, various approaches and countermeasures regarding MUAs are required in the anticipation of such population trends. A geographic information system (GIS) processes and manages data using location information to create maps and perform advanced analyses [16,17]. The GIS has been used in studies that investigate healthcare systems [18,19,20,21,22] such as the distribution of healthcare resources and access to healthcare [23,24,25,26]. Recently, a GIS has also been used in simulation studies to anticipate the shifts in healthcare access based on predictions of future populations and the possible locations of medical institutions [27,28]. To ensure healthcare access and systems in MUAs, this study aimed to identify possible future changes in MUAs in Japan using a GIS-based simulation analysis. This study would provide information for reforming effective policies and actions for MUAs in light of future population trends. This information seems to be useful in other countries with aging populations and depopulation.

## 2. Materials and Methods

### 2.1. Ethics Statements

This study was conducted using a publicly available dataset; personal information was not accessed. Therefore, this study did not require approval from an institutional review board.

### 2.2. Study Design

This study covered all 380,948 1 km meshes, all 87,942 clinics, and all 8354 hospitals in Japan. The 1 km mesh is a grid divided by a latitude of 30 s and a longitude of 45 s. It is called a 1 km mesh because it covers an area of approximately 1 square kilometer. This is a standardized statistical unit in Japan and the most commonly used aggregation unit in national statistics [29]. The 1 km mesh data were obtained from the ArcGIS Data Collection Standard Pack 2023 (ESRI Japan, Tokyo, Japan). This database contained meshes for the 2020 Census. In the analysis, 380,948 1 km meshes were used (excluding 6298 meshes of 1 km for the Northern Territories). Population data for each 1 km mesh-based estimate in 2020, 2030, 2040, and 2050, published by the Ministry of Land, Infrastructure, Transport, and Tourism, were used [10]. Data on clinics and hospitals were obtained from the Institution for Health Economics and Policy [30]. The database comprises data from nationwide medical institutions in Japan published by the Ministry of Health, Labour and Welfare in 2018 [30]. The Institution for Health Economics and Policy added information about the latitude and longitude based on the address of each medical institution to make the data easier to process using a GIS. We plotted all 87,942 clinics and 8354 hospitals included in this database with the GIS using the “XY table to point” command of ArcGIS Pro (ESRI, Redlands, CA, USA). 

### 2.3. Definition and Analysis

**Figure 1:**
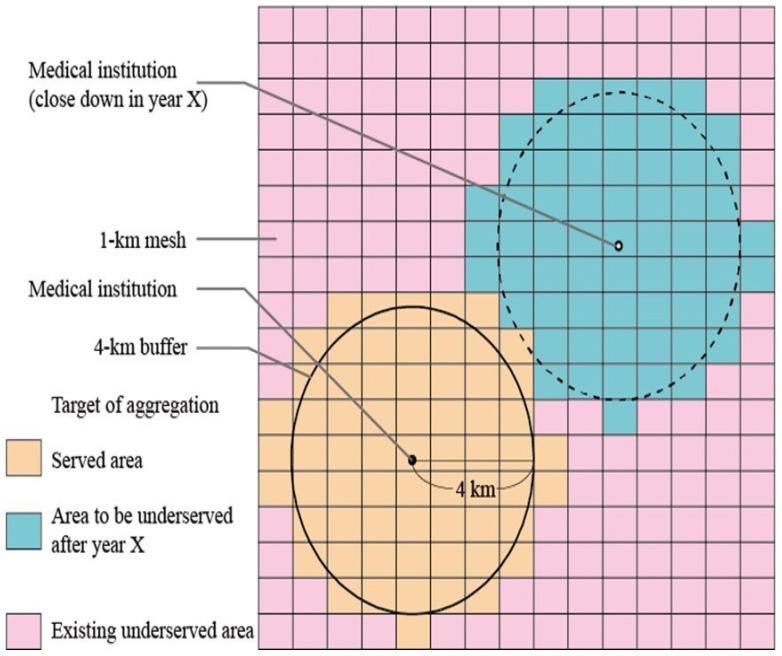
Schematic explanation of areas. A 4 km radius buffer (circle) is generated for each medical institution; 1 km meshes that overlapped the buffer are defined as a medically “served” area, whereas those that did not meet the definition are determined as a medically underserved area (MUA). The MUAs are categorized into areas with populations of 50–999 and <50 residents.

Areas within a 4 km radius of medical institutions were considered medically “served” areas (MSAs) as of 2020, whereas areas outside that definition were considered as MUAs. The 4 km radius denotes the straight-line distance between two points. Therefore, we created a 4 km radius buffer from each medical institution using the buffer command of ArcGIS Pro 3.0 (Figure 1). Areas outside the 4 km radius buffer as of 2020 were designated as existing MUAs. For the 2030, 2040, and 2050 simulations, the target population within a 4 km radius of each medical institution was tabulated for each year. The simulation study assumed that if the target population within a 4 km radius fell below 1000, the medical institution that existed as of 2020 would close (Figure 1). In 2030, 2040, and 2050, MUAs with a target population below 1000 were identified as new MUAs. New MUAs were tabulated for each year. In Japan, the presence of 50 and 1000 persons of the population is the key criteria for the healthcare policy in rural areas because one of the requirements for the designation of NPC is that the population of the area must be 50 or more, and the criterion for the establishment of a rural public clinic is a population of 1000 or more [11,12,13,14,15]. Therefore, new MUAs were classified according to the population size as follows: those with 50 or more but fewer than 1000 people and those with fewer than 50 people. A distribution map was created using the numbers of MSAs, new MUAs, and existing MUAs for each year. All calculations, including the analysis of geographic information, were performed using ArcGIS Pro 3.0.

## 3. Results

### 3.1. Population Trends 

**Figure 2:**
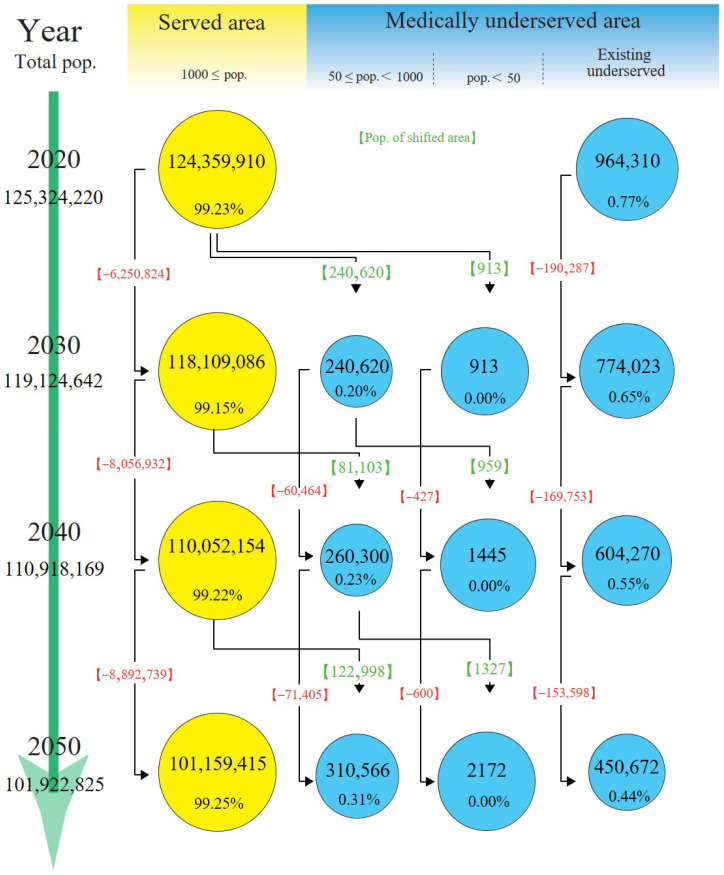
Population trends. Populations are presented as numbers and percentages for every 10 years. The circle sizes reflect the degree of change in the population in each area relative to 2020. Red values indicate net changes in populations. Green values indicate the population in the newly shifted areas from medically served to medically underserved areas. pop.: populations.

The population of MSAs was predicted to decrease from 124,359,910 in 2020 to 101,159,415 in 2050, whereas the proportion of the total Japanese population would remain the same at approximately 99.2% (Figure 2). The population of MUAs was predicted to decrease from 964,310 in 2020 to 763,410 in 2050, whereas the proportion of the total Japanese population would remain the same at approximately 0.8%. Additionally, the population in existing MUAs was predicted to decrease by more than half from 964,310 to 450,672. During the same period, 310,566 new MUAs with 50 or more but fewer than 1000 residents and 2172 new MUAs with fewer than 50 residents were predicted to be added to previously served areas.

### 3.2. Land Area Trends

**Figure 3:**
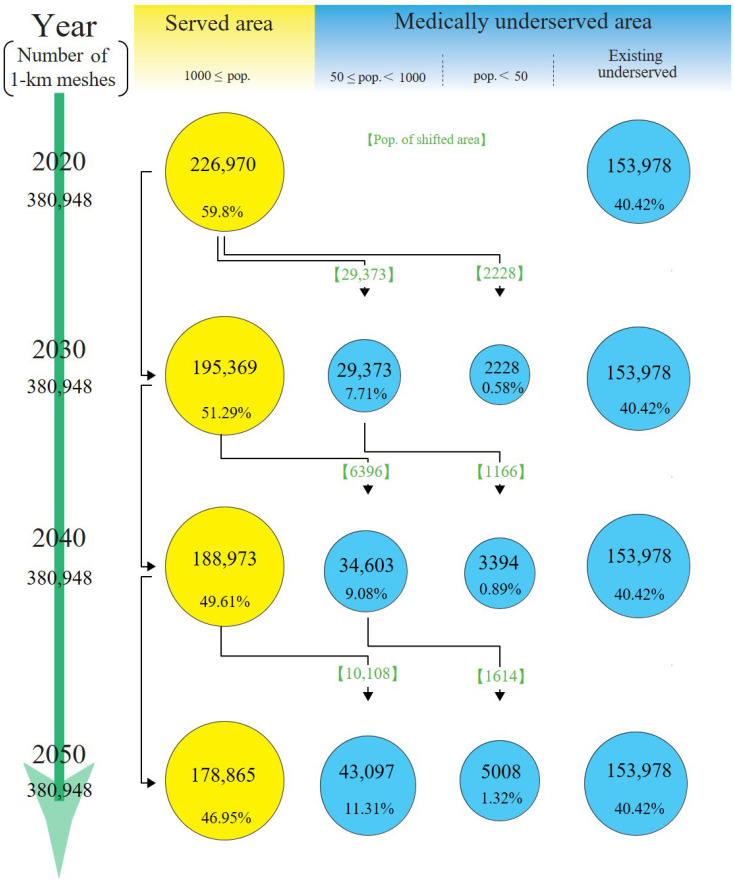
Land area trends. Land areas are presented in 1 km meshes every 10 years. The circle sizes reflect the degree of change in the land area in each area relative to 2020. Green values indicate land areas in newly shifted areas from medically served areas to medically underserved areas. pop.: populations.

**Figure 4:**
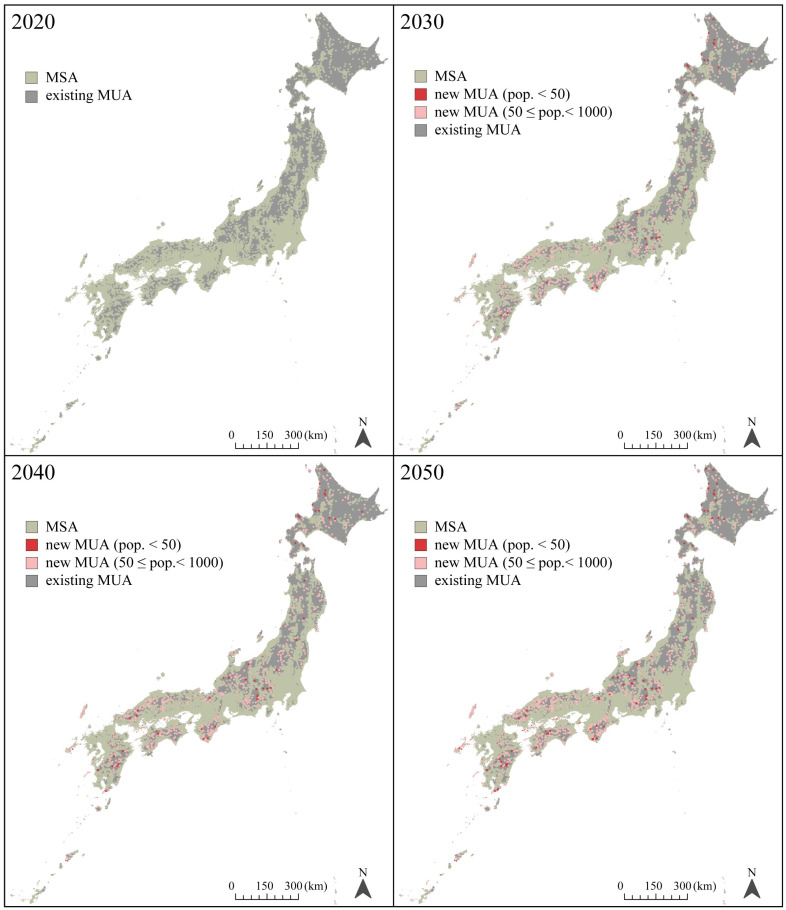
Distribution map of MSAs, new MUAs, and existing MUAs from 2020 to 2050. Land areas are presented in 1 km meshes every 10 years. MSA: medically served area, MUA: medically underserved area, pop.: populations.

In 2020, the land areas of MSAs comprised 59.6% of the total land area of Japan, and those areas were predicted to decrease to 47.0% by 2050 (Figure 3). By 2050, 48,105 meshes (13% of the total 1 km meshes in Japan) were predicted to be new MUAs. The number of MUAs was predicted to increase by 31.2% between 2020 and 2050. In the 48,105 meshes, the number of new MUAs with 50 or more but fewer than 1000 residents were 43,097, and the number of new MUAs with fewer than 50 residents was 5008. The land area of existing MUAs remained at 40.4%. Figure 4 illustrates the distribution map of MSAs, new MUAs, and existing MUAs from 2020 to 2050. New MUAs were located in mountainous areas rather than coastal areas. 

### 3.3. Estimated Number of Medical Institutions

In 2020, there were 96,296 medical institutions. Between 2020 and 2030, 1128 medical institutions were estimated to close, 174 between 2030 and 2040, and 299 between 2040 and 2050. Between 2020 and 2050, 1691 medical institutions were predicted to close.

## 4. Discussion

In the GIS-based simulation analysis, future changes were expected in the MUAs in Japan. From 2020 to 2050, the population of MUAs was predicted to decrease by approximately 20%, whereas the number of land areas of MUAs were predicted to increase by approximately 30%. These findings indicate that access to healthcare for residents living in MUAs might be reconsidered as the population decreases, possibly leading to the reform of rural healthcare policy and systems.

The study results may be explained by the “compact scenario”; in a previous study related to population-based simulation analysis, it was expected that the population would be concentrated in areas with a high population density within the municipality as the population decreases; this theory was referred to as the “compact scenario” [31]. In Japan, where the population is decreasing, the concentration of the population in densely populated areas and the closure of medical institutions in underpopulated areas may be the reasons for the increase in MUAs. Such a vision of the future of MUAs is important for planning Japan’s healthcare delivery. Moreover, this vision can be used for the healthcare systems of other countries where depopulation is expected in the future. It will contribute to the formulation of effective policies and measures for MUAs with an eye toward future population trends.

This simulation study estimated that the number of MUAs would increase in 2050 compared with the existing MUAs and that MUAs would expand. Particular attention should be paid to the increasing number of MUAs with populations of 50 or more but fewer than 1000 among new MUAs because areas with ≥50 residents have the potential to be designated as NPCs. Owing to progress in ensuring physicians in NPCs and partially improving access to medical care by developing transport systems, the number of NPCs decreased from 2920 in 1966 to 557 in 2022 [11]. However, the present study’s findings indicate that NPCs may increase in the future. Nevertheless, access to medical institutions and physicians could lessen, and more areas would have difficulty accessing healthcare institutions, leading to a greater disparity in healthcare delivery between MUAs and MSAs. Patients living in MUAs may experience negative outcomes because of acute and chronic diseases [32,33,34,35]. Overcoming this disparity might require the reform of healthcare systems. Besides obtaining funding to prevent closures of medical institutions, promoting area management by consolidating resource networks is another idea [36,37]. The application of various services with new technologies, such as telemedicine, patient transport, and mobile clinics, in MUAs is considered to complement access to medical institutions and physicians [4,38,39]. The use of these new technologies could improve healthcare access in MUAs and improve outcomes through better disease management. Furthermore, revising the current definition of NPC is also considered to be a potential idea to reconstruct the distribution of healthcare resources [13,14,15].

Measures for MUAs should not be restricted to the medical conditions in rural areas because depopulations in rural areas and the concentration of populations in urban areas can lead to an increase in MUAs [40,41]. Various measures are being taken in different countries facing the decrease in populations in the areas. For example, in Finland, which is experiencing progressing depopulation, the effect of the expansion of broad infrastructure that enables high-speed Internet prevented the depopulation of rural areas [42]. Since high-speed Internet improves opportunities for rural residents and entrepreneurs to use E-services, telemedicine, and tele-commuting, alleviating the locational disadvantage of rural areas, the expansion of broadband Internet is an element ensuring the community activities of the rural areas [42,43]. In France, the total fertility rate improved, preventing depopulation, due to generous financial support for families raising children and the development of an environment that facilitates both childcare and work [44,45]. In Japan, the establishment of the “Community-based Integrated Care System” is being promoted. This system ensures the provision of health and medical care, nursing care, prevention, housing, and livelihood support for daily living in certain communities at the municipal level [41,46,47]. Diverse components, such as clinics for home medical care, home-visit nursing stations, welfare-related administrative organizations, informal services, and self-help groups, will also be included in the system [41,47]. This system notes that most people live in their familiar community for a long time, reducing concerns that older adults (especially those living alone) will leave their own neighborhoods for medical care reasons [41,46]. Another measure, the Children and Families Agency was established in 2023 to strengthen policies for families raising children and improve the low fertility rate [48]. Additionally, to improve the concentration of the population in urban areas, some local governments in Japan provide financial assistance for those who move from urban areas to a local area [49].

This study has several strengths. First, it used highly reliable results from government statistical data. Second, because this study encompassed all of Japan, the results are representative of the entire country. Nevertheless, this study has some limitations. First, the threshold for the closure of medical institutions was simply assumed to be fewer than 1000 residents within a 4 km radius [13,14,15]. However, these institutions may continue operating in the real world. Second, the MUAs in this study and NPCs do not precisely align. The main reason for this discrepancy is that NPC designation is subject to the discretion of local governments. Although NPCs are defined as having more than 50 residents within a 4 km radius with difficulty accessing medical institutions and physicians [13,14,15], in practice, the difficulty in accessing medical services depends on the judgment of local governments. Despite the MUAs and NPCs not exactly matching, the MUAs identified could potentially be candidates for NPC designation. Third, the transportation infrastructure (e.g., road conditions, railroad systems, and buses) of each area could not be evaluated, so it is subject to future changes.

## 5. Conclusions

This GIS-based simulation study showed a future characterized by a decrease in population and an increase in the land area of MUAs in Japan. These findings suggest that the market in healthcare for residents living in MUAs might shrink as the population decreases, thus ensuring healthcare access may be further needed. This study thus seemed to contribute to the development of various services with technologies, as well as effective policies and measures for MUAs considering future population trends. Moreover, such a vision of the future of MUAs could provide crucial information not only for Japan’s healthcare delivery but also for the healthcare systems of other countries facing aging and depopulation challenges (e.g., Spain, Italy, and Finland). In addition, addressing these issues will require not only medical care measures but also comprehensive strategies to manage sustained low levels of fertility and the concentration of populations in urban areas.

Because this is based on a simple simulation, we should monitor the land area of MUAs, as well as the actual policies and measures in healthcare for MUAs in the future. Future studies are warranted.

## Data Availability

The data supporting the study findings can be accessed through the Ministry of Land, Infrastructure, Transport and Tourism (https://nlftp.mlit.go.jp/ksj/gml/datalist/KsjTmplt-mesh1000h30.html (accessed on 29 November 2023)) and the Institution for Health Economics and Policy (https://www.ihep.jp/publications/other/?y=2018 (accessed on 29 November 2023). ArcGIS data collection standard pack 2023 is available from Esri Japan.
